# Case Report: Ultralow-field portable MRI improves the diagnosis of congenital hydrocephalus

**DOI:** 10.3389/fped.2025.1463314

**Published:** 2025-02-27

**Authors:** Anne Groteklaes, Till Dresbach, Markus Born, Andreas Mueller, Hemmen Sabir

**Affiliations:** ^1^ Department of Neonatology and Pediatric Intensive Care, Children’s Hospital, University Hospital Bonn, Bonn, Germany; ^2^ Division of Pediatric Radiology, Department of Radiology, University Hospital Bonn, Bonn, Germany

**Keywords:** newborn, low-field portable MRI, tumor, hydrocephalus, aqueductal stenosis, metastasis, point-of-care

## Abstract

**Introduction:**

Congenital hydrocephalus is an increasing condition both in high as in low and middle income countries. Main causes include aqueductal stenosis, neonatal central nervous system infections, intracranial hemorrhage, malformations and tumors. Investigation of its etiology should include magnetic resonance imaging (MRI) to detect especially pathologies of the fossa cranii posterior. However, MRI is not available to every infant presenting with congenital hydrocephalus especially in those countries with the highest prevalence. New portable ultralow-field MRI (ULF) allows low resource and bedside imaging and thus widens the access to MRI for those infants. This study presents two cases of newborns with congenital hydrocephalus who underwent ULF scanning revealing a tumor of the fossa cranii posterior as cause of hydrocephalus. This study shows that ULF scanning allows to detect and characterize brain tumors as well as metastases.

**Setting and patients:**

In this case report, we present two cases of newborns antenatally diagnosed with hydrocephalus with no further pathology detected in repeated cranial ultrasound and, in one case, fetal MRI. We performed ULF imaging using a portable 0.064T MRI during natural sleep and high-field 3T MRI to investigate the etiology of congenital hydrocephalus in these infants.

**Main results:**

ULF imaging revealed a tumor of the fossa cranii posterior in both cases. MRI signalling detected in ULF imaging was specific for each tumor (ATRT, low grade glioma). In one case, ULF imaging also detected intracerebral metastasis.

**Conclusions:**

We demonstrated that ULF imaging is able to detect tumors of the fossa cranii posterior that are not detected on ultrasound and shows their specific MR-signalling as well as detect metastasis. Additionally, compared to 3T MRI, ULF MRI was able to reveal significant findings while requiring fewer resources and being easier to perform. Therefore, we propose that children with congenital hydrocephalus not showing any abnormalities on cranial ultrasound should undergo ULF MRI. This imaging modality holds potential for monitoring neonatal tumors and detecting metastasis.

## 1 Introduction

Congenital hydrocephalus is the most common indication for neurosurgical interventions in neonates. Its incidence has significantly increased in low- and middle-income countries (LMIC), with an incidence of 123/100.000 live births (1–4). The most common causes of congenital hydrocephalus are aqueductal stenosis, neonatal central nervous system infections, intracranial hemorrhage, and malformations, such as neural tube defects or tumors (5). Fetal ventriculomegaly is one of the most commonly diagnosed abnormalities in prenatal ultrasound scanning (1–2/1,000 live births) (6).

While fetal ventriculomegaly is usually diagnosed with ultrasound, this technology has several limitations, as it is operator-dependent and the ability to penetrate the fetal calvarium is limited. In particular, pathologies in the fossa cranii posterior are often missed when performing only ultrasound imaging. Magnetic resonance imaging (MRI) can detect additional anomalies such as cerebellar tumors, anatomic abnormalities, cysts, and hemorrhages in 20%–50% of the cases with no additional findings on ultrasound, especially in corpus callosum pathologies, hemorrhage, folding abnormalities, and other brain abnormalities (7–9). However, not every infant presenting with congenital hydrocephalus receives MR-imaging, and especially in countries with the highest prevalence (LMIC), these resources are very limited (10–12). The reason for this is the high resource requirement characterized by high costs and the need for technical, spatial and personnel infrastructure such as reliable-continuous high-power electricity supply, liquid helium which maintains the superconductivity of the magnet, electromagnetic shielding, and a large fringe field safety exclusion radius (13).

Ultralow-field portable MR Imaging (ULF) is a new imaging tool that allows low-resource and bedside imaging. The portable 0.064-T MRI device currently available (Hyperfine, Gulford, CT) has a magnetic field formed by two horizontally oriented permanent magnets, while the receiver coil and radiofrequency transmit coils are on a platform inside the gantry (14). ULF imaging requires fewer resources owing to its lower cost and lower personnel and infrastructure requirements. Moreover, it reduces the risks, as patients do not need to be transported or sedated for imaging (15, 16). ULF MR-devices have already been trialed in both adult and pediatric settings (15–18, 19–21). Thus, ULF provides a new opportunity to provide MRI to infants with congenital hydrocephalus who would not otherwise receive an MRI, both in LMIC (due to limited resources) as well as in high-income countries, as not every infant presenting with congenital hydrocephalus receives an MRI (9).

Herein, we present two cases of newborns antenatally diagnosed with fetal hydrocephalus due to presumed aqueductal stenosis. As no other pathologies were found on initial postnatal cranial ultrasounds, both infants underwent ULF imaging to detect a possible cause of the hydrocephalus. In both infants, a tumor of the posterior fossa was found. This diagnosis makes a significant difference in the treatment and prognosis of these infants. Findings were missed in repeated cranial ultrasounds by experienced operators and could be clearly diagnosed using ULF imaging. Moreover, ULF imaging detected metastases in one infant that were not detected on cranial ultrasound.

## 2 Patient information

### 2.1 Case 1

A girl was born at 36 + 6 weeks of gestation (WOG) by cesarean section to a 35-year-old mother. Prenatal ultrasound performed in 32 WOG showed ventricular hydrocephalus due to assumed aqueduct stenosis.

### 2.2 Case 2

A boy was born at 37 + 6 WOG via cesarean section to a 36-year-old mother. Prenatal ultrasound performed at 23 WOG showed fetal hydrocephalus with mild dilatation of the third ventricle.

## 3 Diagnostic assessment

### 3.1 Ultralow-field and standard imaging

**Figure 1:**
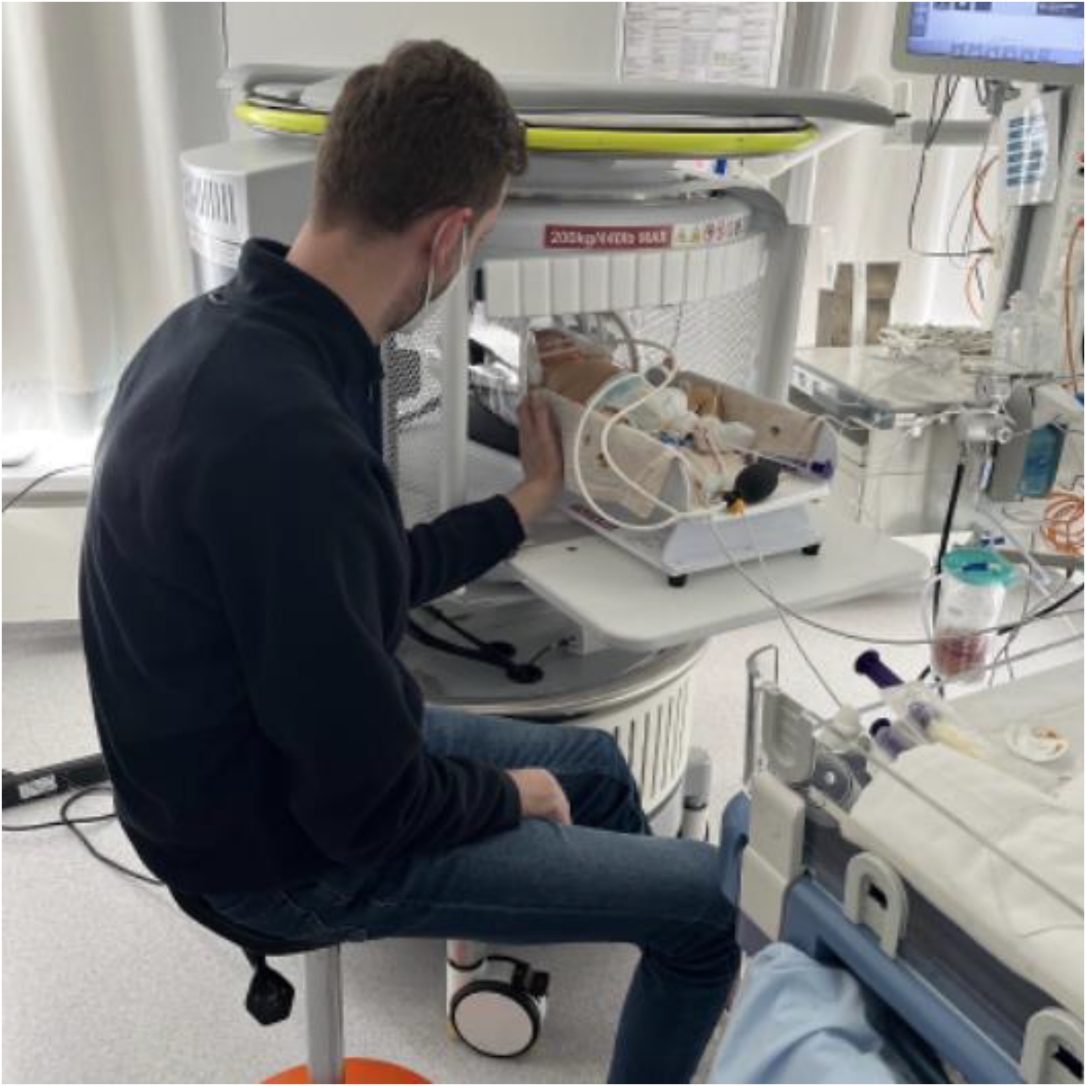
Setting of bedside ULF scanning. Infants were scanned on the Hyperfine Inc. Swoop system, equipped with the single channel transmit/eight channel receive adult head coil with additional Hyperfine Inc. Baby Nest which places the infant's head at the magnet isocentre. Parents could touch their infant during the ULF scan.

Infants underwent ULF scanning on a 0.064T (64mT) Swoop® (Hyperfine, Inc., Guilford, USA; hardware version: 1.7, software version 8.7 beta) utilizing the built-in radiofrequency interference rejection method, 29 single-channel transmit/eight-channel receiver adult head coil with additional Hyperfine Inc. Baby Nest, designed to facilitate the imaging of newborns by placing their head at the magnet isocentre. The setting during the ULF scan is shown in Figure 1.

After parental consent infants were scanned at their bedside, either just next to the mother bed at the delivery station, or at their cot in the neonatal ward. The local ethics committee approved the study to be performed at the Children's Hospital, University Hospital Bonn/Germany (Ethics Nr 167/22). Parents and infants could stay with each other during the entire scan, and parents could also touch their baby to not disturb the early time of parent-child interaction. The infants did not receive any sedation, and imaging took place during natural sleep. A pediatrician supervised the clinical care of all infants during the imaging. The sequences acquired were T1 (TI 500 ms, UNITY), T2 (axial and isotropic imaging, 2 mm linear scan), axial FLAIR, and axial DWI (b = 0 s/mm^2^ and b = 900 s/mm^2^). The scanning time was approximately 48 min. Images were reconstructed by the system using linear image reconstruction methods (including geometric distortion correction and receive coil sensitivity correction. Images were post-processed using Python (Dicom2niftie), FLIRT, and Fsllab. We acquired at least 2 T2-sequences which were then averaged using FSLlab. The only shielding utilised by the Hyperfine Swoop is radio frequency electromagnetic shielding, which is achieved by the use of a partial faraday cage. The entrance to the scanner is an opening in this cage, which the patient is placed through and the parent can reach through to settle their child. There is no static magnetic field shielding employed, however the Gauss Guard indicates the extent of the 5 Gauss Line. Parents are screened for MRI safety using an MRI screening form before entering this zone, as are patients. ULF MRI was performed by a pediatrician. No other personnel was involved for the scan.

Standard MRI neuroimaging was performed using a 3T Philips Ingenia Elition scanner. The infants were imaged using an MR incubator and sedated by an experienced clinician. Personnel included involved: The neonatologist performing the sedation, a nurse attending a scan, another nurse helping for the transport of the infant from the neonatal ward to the radiologic department, a radiologist and a radiological technical assistant. The sequences acquired were T1, T2, SWI, DWI, and contrast-enhanced. The scanning time was ∼90 min.

Cranial ultrasound was performed with a Philips AA26050 L ultrasound machine. Prenatal ultrasound was performed using a GE ultrasound machine by DEGUM III operators.

Fetal MRI was performed at a 1.5T Philips Ingenia scanner and at a 1.5T Siemens Solar scanner.

#### 3.1.1 Case 1

Repeated fetal ultrasounds showed no further intracranial pathologies, and screening for congenital infectious diseases [cytomegalovirus (CMV), Toxoplasma gondii, and parvovirus B19] was negative. No fetal magnetic resonance imaging (MRI) was performed.

**Figure 2:**
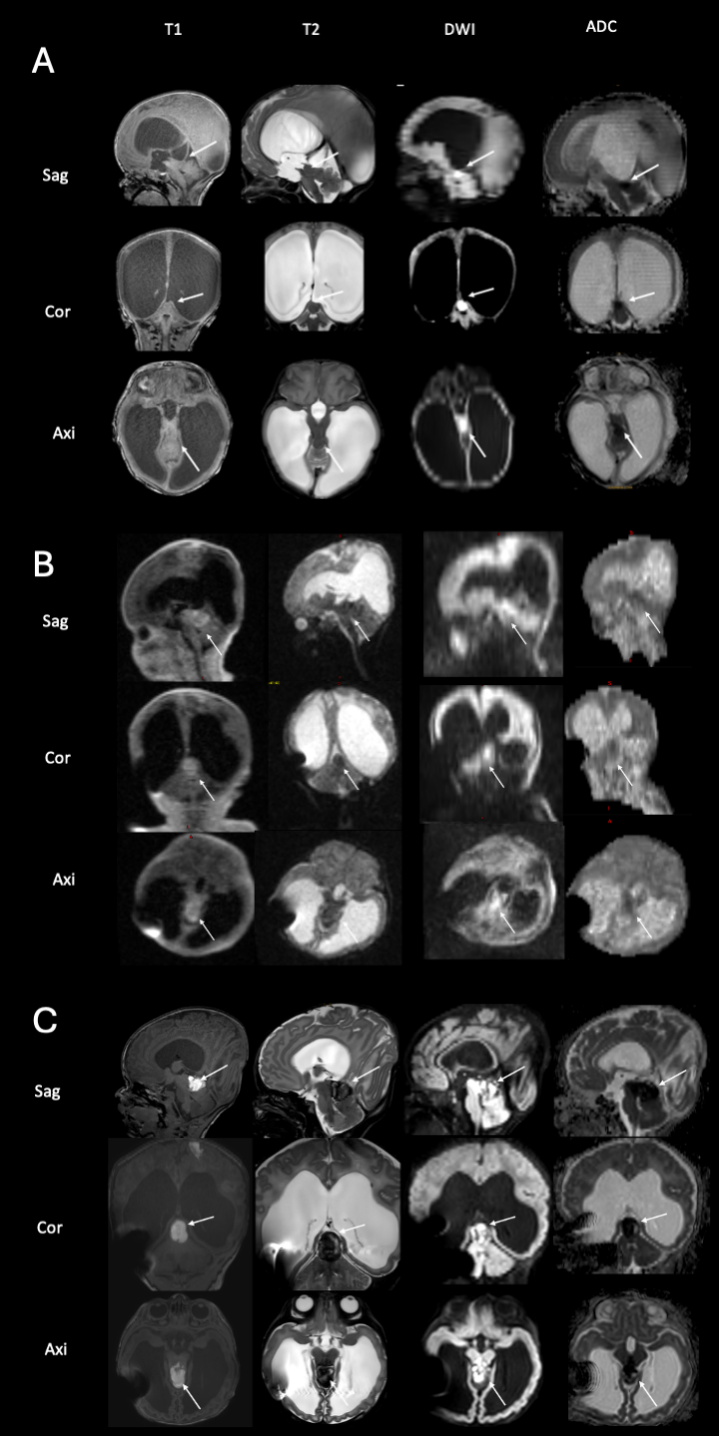
Imaging of the tumor in the fossa cranii posterior of case 1 with high-field imaging at 5th day of life **(A)**, ultralow-field imaging at 11th day of life **(B)** and high-field imaging at 19th day of life **(C)** the scan at 5th day of life reveals a tumor of the fossa cranii posterior (arrows) with low T2 and cortexisointense T1-signalling and diffusion restriction **(A)** the ULF scan at 11th day of life **(B)** shows the tumor has hemorrhaged (visible in T1, arrows) and confirms the characteristical signalling of ATRT tumors with low T2 signal and diffusion restriction. Note the artifact by the implanted VP shunt. The high-field scan at 19th day of life **(C)**, confirms the findings of the ULF scan (compare to B).

Postnatally, no additional findings were found on cranial ultrasonography. As the head circumference increased, surgery was planned on the 5th day of life. Prior to surgery, the infant underwent standard high-field 3 Tesla MR scanning, which revealed a tumor of the fossa cranii posterior (Figure 2A, arrows).

**Figure 3:**
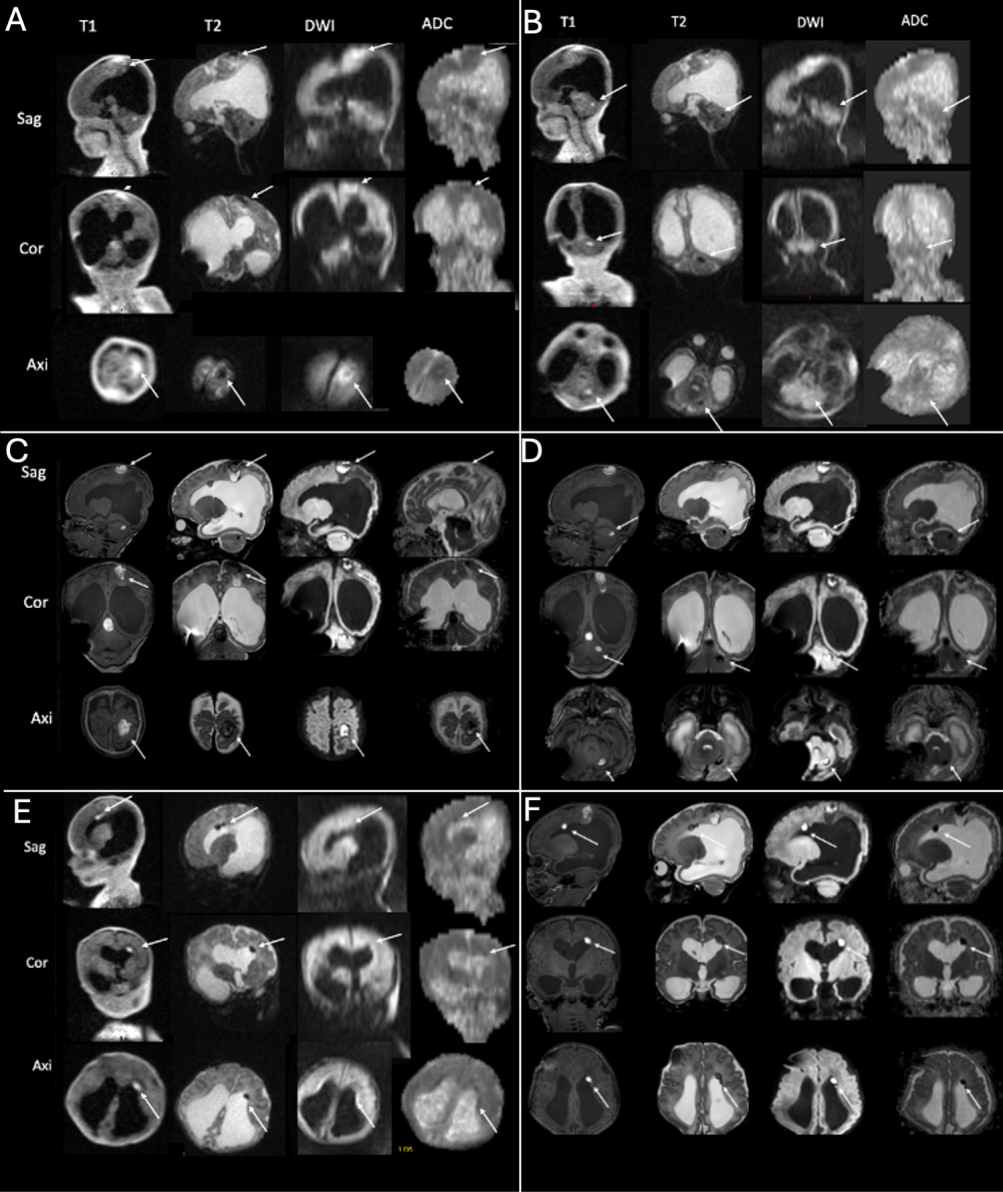
ULF **(A,B,E)** and high-field **(C,D,F)** imaging of case 1. The ULF scan at 11th day of life reveals parietal [**(A)** arrows], cerebellar [**(B)** arrows] and periventricular [**(E)** arrows] metastasis with a high T1 and low T2 signal and diffusion restriction. The high-field scan at 19th day of life confirms the findings of the parietal [**(C)** arrows], cerebellar [**(D)** arrows] and periventricular [**(F)** arrows] metastasis).

Shunt surgery was performed without taking any tissue samples, and 7 days later (on the 11th day of life), ULF imaging was performed confirming the pre-known tumor and detecting it had hemorrhaged since the time the HF-MRI was performed (Figure 2B, arrows). The mass in the tumor showed massive diffusion restriction and a cortex-isointense T2 signal (Figure 2B, arrows). In addition, the ULF scan detected new disseminated metastases supra- and infratentorially, showing a high T1 signal and diffusion restriction on DWI and a corresponding low T2 signal (Figures 3A,B,C, arrows). 3T MRI was performed on the 19th day of life and confirmed the findings of the ULF MRI (Figures 2C, 3C,D,F, arrows). In knowledge of the MR findings, we performed targeted cranial ultrasound by experienced operators, which also confirmed the findings of the ULF scan. Biopsy was taken at 21th day of life.

Taken together, ULF MRI showed a tumor of the fossa cranii posterior with low T2 and cortexisointense T1-signalling and diffusion restriction (Figure 2B, arrows). This is a characteristical signalling of atypical teratoid rhabdoid tumor (ATRT) with low T2 signal and diffusion restriction. Moreover, ULF MRI revealed parietal (Figure 3A, arrows), cerebellar (Figure 3B, arrows) and periventricular (Figure 3E, arrows) metastasis with a high T1 and low T2 signal and diffusion restriction.

Taken together, due to its typical radiologic features and rapid metastasis, a diagnosis of ATRT was suspected. The best supportive care was offered, and the infant went home with his parents.

#### 3.1.2 Case 2

Repeated fetal ultrasound by DEGUM III operators showed a ruptured midline with no further intracranial pathologies, screening for CMV and Toxoplasma Gondii was negative, genomic testing including trisomy 13,18,21, and trio exome analysis from amniocentesis showed no pathologic findings. Fetal MRI was performed at 26 + 5 and 34 WOG, showing features of aqueduct stenosis.

Postnatally, no additional findings were found on cranial ultrasonography by several investigators.

**Figure 4:**
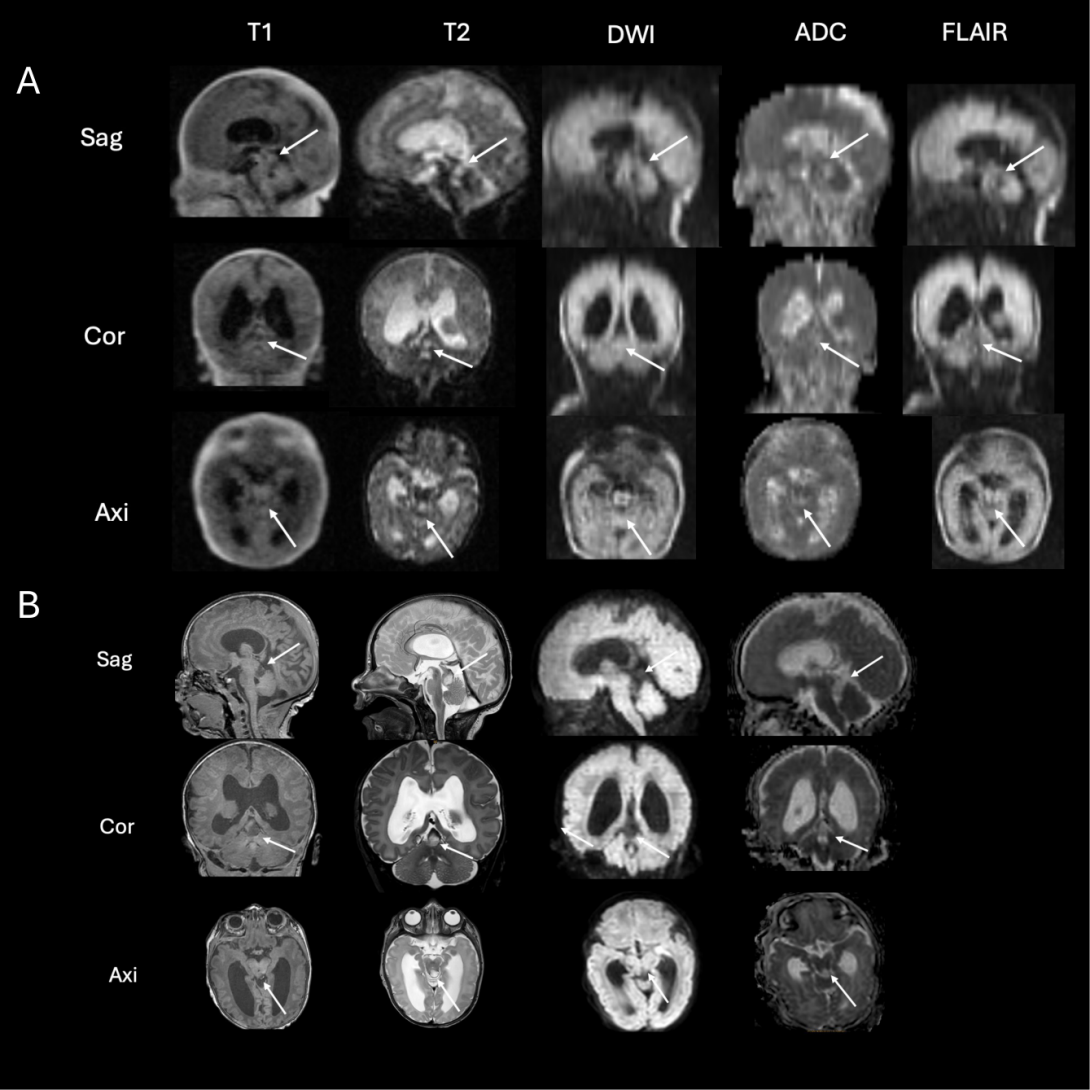
ULF **(A)** and high-field **(B)** scanning of case 2. The ULF scan at 4th day of life reveals a tumor of the fossa cranii posterior [**(A)** arrows] showing high T2 signal and low T1 signal without diffusion restriction. The high-field scan at 5th day of life **(B)** confirms the ULF finding from the previous day showing a tumor of the fossa cranii posterior (arrows) with low T1 and high T2 signal without diffusion restriction.

The infant underwent ULF scanning on the 4th day of life, revealing a tumor of the fossa cranii posterior (1.1 × 1 × 0.8 cm) (Figure 4A, arrows). The tumor showed a high T2 signal and low T1 signal (Figure 4A, arrows). The tumor did not show any diffusion restriction (Figure 4A, arrows). These findings were confirmed by 3-T-MRI on the 5th day of life (Figure 4B, arrows) as well as by afterwards performed targeted cranial ultrasound by experienced operators in knowledge of the MR-findings. As the head circumference increased, surgery was planned on the 6th day of life.

Taken together, this tumor showed typical radiologic features of low-grade gliomas (high T2 signal, low T1 signal, no diffusion restriction as seen in Figure 4). To date, no biopsy has been performed. The infant then underwent active surveillance. A second MRI, performed in the 6th week of life, showed no progression.

## 4 Discussion

In this study, we present two neonates born with congenital hydrocephalus with no known additional pathologies on repeated ultrasound scans and, in one patient, two fetal MRIs. ULF scanning revealed tumors of the fossa cranii posterior as the underlying etiology for hydrocephalus and, therefore, made a huge change to therapeutic procedures as well as prognosis. Moreover, we showed that ULF can be used to detect metastasis in the neonatal brain. This is the first reported case of a neonatal tumor and metastasis detected using ULF scanning. Moreover, we showed that ULF scanning can not only detect brain tumors, but also show specific radiologic features of brain tumors (in this case shown for low-grade glioma and ATRT) and therefore has a high influence on the treatment of these children and even the decision to go for best supportive care.

The aqueductal stenosis is a common cause of congenital hydrocephalus (6). Its etiology can be due to an anatomic anomaly as well as a secondary change due to various pathologies, such as central nervous system infections (CMV, Toxoplasmosis) and subsequent adhesions, or external compression through tumors, cysts, or intracerebral hemorrhage as well as genetic reasons (5, 22, 23). Therefore, the diagnosis of fetal ventriculomegaly should be accompanied by testing for infections (CMV, Toxoplasmosis) as well as genetic testing (5, 24). Radiographic evaluation should include ultrasonography to further investigate the cause of hydrocephalus. Fetal MRI is used to provide information about the etiology of hydrocephalus and to identify additional anatomic anomalies, such as septo-orbic dysplasia and malformations of cortical development, leading to closer observation, monitoring, or further interventions (5–7, 24–26).

Postnatally, routine surveillance should include physical examination, serial head circumference measurements, and serial radiographic imaging (5). Radiographic examinations with cranial ultrasound should be performed to provide further information on the etiology and surveillance of infants (6). Postnatal MRI is required to investigate the etiology of hydrocephalus, as well as the appearance of the ventricular system, which may significantly change surgical strategies (5).

ULF portable MRI is a new technique that allows bedside imaging in both high- and low-resource settings. ULF imaging requires less resources, due to its lower cost, and lower personnel and infrastructure requirements. Moreover, it reduces the risks as patients do not need to be transported or sedated for imaging (27). Thus, ULF MRI provides a new opportunity to provide MRI to infants with congenital hydrocephalus who would not receive an MRI otherwise, both in LMIC as well as in high-income countries. Moreover, ULF MRI could allow point-of-care imaging within high-resource environments. There is increasing evidence of using ULF MRI in diagnosing different diseases, so far primarly described in the adult population (11–17). For example, several studies have shown the possibility to detect and monitor strokes in adult patients (15, 28–30). There is limited evidence in children and neonates. However, it has been shown that ULF MRI can be used in ECMO patients (16), in neonates detecting hydrocephalus, hypoxia- ischemia and agenesia of the corpus callosum (15, 20) and in children detecting intracranial hemorrhage (31). In adults, detection of intracerebral tumor using ULF MRI has been described in few cases (10, 32–34); however, no detection of metastasis using ULF MRI has so far been reported in literature and this is the first description of diagnosis of metastasis and ATRT using ULF MRI.

In this study, we demonstrated that ULF MRI is safe and feasible in neonates with congenital hydrocephalus. We showed that ULF imaging can be performed without the use of sedation and without separating the parents from their children at the bedside in neonatal and delivery wards. We showed that there were no restrictions on medical care owing to ULF imaging. Moreover, in one case, we demonstrated that ULF imaging can be performed in children with a ventricular-peritoneal (VP) shunt and that shunting positions have not changed due to ULF MRI. Therefore, ULF MRI can be used as a safe diagnostic tool for infants with VP shunts.

We demonstrated that ULF scanning can detect intracerebral tumors that had not been detected on either fetal MRI or sequential pre- and postnatal cranial ultrasound. We showed that ULF MRI could detect intracerebral metastases. To the best of our knowledge, these are the first cases described in the literature with ULF scanning for the detection of neonatal tumors and metastasis. We demonstrated that ULF scanning can not only detect these findings but also show specific radiologic features of brain tumors (in this case, ATRT and low-grade glioma), and therefore lead to therapeutic decisions.

Comparing ULF imaging with conventional high-field imaging, high-field scanning has a higher resolution and shows a more detailed image of the brain. Moreover, contrast enhancement can be used on high-field MRI, which has so far not been trialed in ULF, and therefore provides more information about the tumor. Thus, there are limitations of the use of ULF MRI for the diagnosis of congenital hydrocephalus, as especially very small pathologies require higher resolution and higher image quality than ULF can so far provide. Nevertheless, even without contrast enhancement, ULF could detect metastasis and show radiologic features typical for specific brain tumors, which were then confirmed by high-field scanning.

As ULF scanning requires fewer resources and, due to its feasibility at the bedside, is less stressful to patients than high field scanning (13, 16), we suggest that all children with congenital hydrocephalus, especially those with aqueductal stenosis and no clear etiology on cranial ultrasound, should be examined with ULF. Moreover, it reduces stress to the parents, as they do not need to be separated from their child, and early parent-child interaction is not disturbed. Patients have not to move significantly, which also reduces stress to the patients as equipment and staff resources, and allows imaging in very unstable patients (15, 16). No extra monitoring is needed, which also contributes to minimizing stress and resources.

To conclude, ULF MRI widens the access of MRI to infants both in high- and low- and middle-income countries who would not otherwise receive an MRI.

We also demonstrated that the ULF characterizes intracerebral tumors and detect metastasis. As it is easy to perform and requires only low resources, it can be used as a serial imaging tool to image neonates with tumors to detect metastasis, progression, and hemorrhages. Furthermore, it is not dependent on the operator's skills and has this advance compared to cranial ultrasound, by which in these cases the findings were missed until knowledge of the tumor was aquired through ULF imaging. Moreover, as it shows specific findings for specific tumors, it helps to make a diagnosis and treatment decisions.

## 5 Patients perspective

Both ULF scans were attended by a pediatrician and both parents. Neither the pediatrician nor the parents noticed any signs of stress in the infants. The parents reported the ULF scan was less stressfull than the high field scan. Both families agreed that ULF scanning was a less invasive opportunity to examine their children.

## 6 Conclusions

Ultralow-field MRI can be instrumental in clarifying the etiology of congenital hydrocephalus, particularly in detecting tumors that may not be detected by fetal MRI and serial ultrasound, even when performed by highly skilled professionals. This is the first reported case of a neonatal tumor and metastasis detected using ULF scanning.

ULF MRI is capable of detecting intracerebral tumors, identifying specific tumor features, and thus aiding in more accurate diagnoses. It can also reveal metastasis, progression, and hemorrhage in neonatal tumors.

Additionally, compared to 3T MRI, ULF MRI was able to reveal significant findings while requiring fewer resources and being easier to perform. Therefore, we recommend that children with congenital hydrocephalus who show no abnormalities on cranial ultrasound should undergo ULF MRI. This imaging modality holds potential for monitoring neonatal tumors and detecting metastasis.

Future research and development are needed for ULF imaging, especially as imaging technologies are rapidly advancing and ULF imaging capabilities improve each year. This progress could make ULF MRI a viable screening and surveillance tool in high-income countries and extend MRI accessibility to low- and middle-income countries. Investigating contrast enhancement in ULF MRI is crucial for further characterizing intracranial tumors.

## Data Availability

The original contributions presented in the study are included in the article/Supplementary Material, further inquiries can be directed to the corresponding author.
